# Supplementary material for: Development of a ‘universal-reporter’ outcome measure (UROM) for patient and healthcare professional completion: a mixed methods study demonstrating a novel concept for optimal questionnaire design
Source: BMJ Open. 2019 Aug 24;9(8):e029741. doi: 10.1136/bmjopen-2019-029741 (PMC6720518; doi:10.1136/bmjopen-2019-029741)
Supplement: Supplementary data [file bmjopen-2019-029741supp001.pdf]

Supplementary file 1: Items for assessing surgical site infection (SSI) in the version of the UROM after pre-testing*

Item	
1	Was there redness spreading away from the wound? (erythema/cellulitis)
2	Was the area around the wound warmer than the surrounding skin?
3	Was any part of the wound leaking fluid?
	a) Was it clear fluid? (serous exudate)
	b) Was it blood-stained fluid? (haemoserous exudate)
	c) Was it thick and yellow/green fluid (pus/purulent exudate)
4	Have the edges of any part of the wound separated/gaped open on their own accord? (spontaneous dehiscence)
	a) Did the skin separate?
	b) Did the deeper tissue separate?
5	Has the area around the wound become swollen?
6	Has the wound been smelly?
7	Has the wound been painful to touch?
8	Have you had, or felt like you have had, a raised temperature or fever? (fever >38°C)
9	Have you sought advice because of a problem with your wound, other than at a routine planned follow-up appointment?
10	Has anything been put on the skin to cover the wound? (dressing)
11	Have you been back into hospital for treatment with a problem with your wound?
12	Have you been given antibiotics for a problem with you wound?
13	Have the edges of your wound been deliberately separated by a doctor or nurse?
14	Has your wound been scraped or cut to remove any unwanted tissue? (debridement of wound)
15	Has you wound been drained? (drainage of pus/abscess)
16	Have you had an operation under general anaesthetic for treatment of a problem with your wound?

Footnote: Items shown here are written in first-person context for patient completion

*Selected text only is included in this table for demonstration purposes and does not show the full list of items/response categories that were included to collect resource use data for the purpose of the case study. Further minor changes have since been made to the numbering and formatting of the items following a validation study
